# Advances in the Metabolic Mechanism and Functional Characteristics of Equol

**DOI:** 10.3390/foods12122334

**Published:** 2023-06-10

**Authors:** Yining Gong, Jiaping Lv, Xiaoyang Pang, Shuwen Zhang, Guofang Zhang, Libo Liu, Yunna Wang, Chun Li

**Affiliations:** 1 Key Laboratory of Dairy Sciences, Ministry of Education, College of Food Science, Northeast Agricultural University, Harbin 150030, China; 2 Institute of Food Science and Technology, Chinese Academy of Agricultural Sciences, No. 2 Yuan Ming Yuan West Road, Beijing 100193, China; 3 Heilongjiang Green Food Science Research Institute, Harbin 150030, China

**Keywords:** equol, soy isoflavone, equol-producing bacteria, application, synthesis technique

## Abstract

Equol is the most potent soy isoflavone metabolite and is produced by specific intestinal microorganisms of mammals. It has promising application possibilities for preventing chronic diseases such as cardiovascular disease, breast cancer, and prostate cancer due to its high antioxidant activity and hormone-like activity. Thus, it is of great significance to systematically study the efficient preparation method of equol and its functional activity. This paper elaborates on the metabolic mechanism of equol in humans; focuses on the biological characteristics, synthesis methods, and the currently isolated equol-producing bacteria; and looks forward to its future development and application direction, aiming to provide guidance for the application and promotion of equol in the field of food and health products.

## 1. Introduction

Soy is one of the crops that are rich in soy isoflavones and is consumed worldwide; it also makes up an important part of traditional Asian diets such as tofu, soy milk, natto, and miso [1]. Most experimental animals currently produce equol when fed soy-containing feed, especially rodents. Equol was first isolated from pregnant mares by Marrian et al. [2]. It was named equol because it was detected in horse urine and belongs to the diphenol with an estrogenic effect. Then, Bennetts et al. [3] discovered that equol is derived from soy isoflavone (SI), proving that equol is an important metabolite. In 1982, Axelson et al. discovered equol in human urine, and the metabolic mechanism of equol has been widely studied since then [4]. Equol has higher biological activity than its precursor compound, daidzein, because it mediates many of its biological effects by binding to estrogen receptors [5], with potential efficacy in regulating both hormone levels and oxidative stress, suggesting that exposure to this compound may have an impact on cancer risk. However, the results of in vitro studies may be influenced by whether R- or S-equol or racemic mixtures are used. A series of in vitro and in vivo studies have shown that equol has a strong anticancer effect, which can inhibit the development of hormone-dependent malignancies such as breast and prostate tumors, as well as hormone-dependent tumors such as colorectal cancer and liver cancer [6]. In addition, topical application of racemic equol has been shown to reduce the proportion of tumors progressing from benign to malignant squamous cell carcinoma [7].

However, there are significant differences in equol production capacity, and only 30% to 60% of people in humans produce equol after the ingestion of soy [8]. People who cannot produce endogenous equol need to meet their own needs through exogenous supplementation of equol, which is low in yield and expensive. Therefore, the fast and efficient preparation technology of equol has become an important research topic. The genera *Lactobacillus*, *Enterococcus*, and *Lactococcus* are a heterogeneous group of lactic acid bacteria (LAB) that have also been proposed as probiotics and as microbial cell factories for the production of nutraceuticals [9]. It is possible to use *Bifidobacterium*, *Lactococcus*, or *Lactobacillus intestinalis* with daidzein to produce equol as food ingredients and supplements. The metabolic mechanism and equol synthesis pathway of known equol-producing bacteria are reviewed in this paper in order to provide a theoretical basis for the synthesis of equol in vitro and its application. This paper details a description of equol metabolism in vivo and in vitro, as well as the functional characteristics and applications of equol.

## 2. Physicochemical Properties of Equol

Equol is a metabolite of daidzein produced by specific gut microbiota in the small intestines and distal part of the colon [10]. The equol molecule is composed of C_14_H_15_O_3_ and its molecular weight is 242.27, which is a heterocyclic structure containing two active hydroxyl groups and a relatively inactive oxygen in its central furan ring [11]. The chemical name of equol is 7-hydroxy-3-(4-hydroxyphenyl)-chroman, and it belongs to nonsteroidal estrogens. Physicochemically, it is a white-to-beige powder with no polarity that is difficult to dissolve in water, unstable in acid, and easily destroyed. It has a melting point that is approximately 189~190 °C [4,11]. Because there is an asymmetric carbon at the C-3 position of the molecule, equol has two corresponding isomers: S-equol and R-equol. S-equol is a natural noncorresponding isomer produced by bacteria in the intestine, while the synthetic compound is a mixed racemic compound of S-equol and R-equol.

## 3. Metabolism and Absorption of Equol

### 3.1. Metabolism and Regulatory Mechanism of Equol

**Figure 1:**
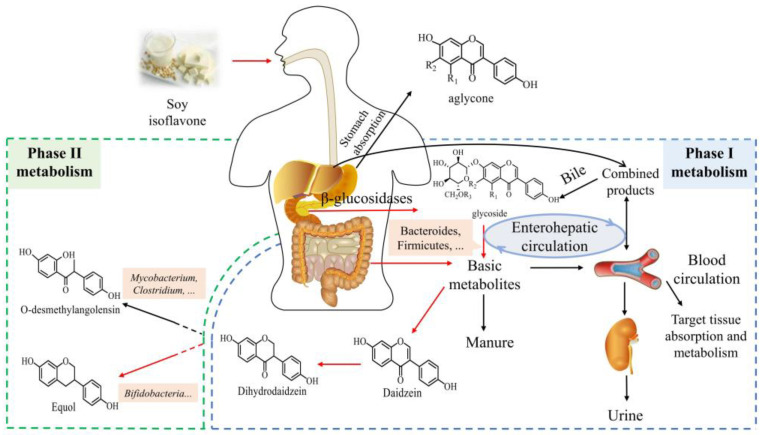
Schematic diagram of the metabolism of soy isoflavones in vivo [17,18].

Foods rich in soy isoflavones are good sources of equol [12]. Soy isoflavones are a class of phytoestrogens widely found in legumes that ultimately form equol by producing the primary metabolite daidzein. Up until now, 12 kinds of soy isoflavones have been discovered which can be divided into two types [13]: free aglycones and bound glycosides, including three free aglycones of daidzein, genistein, and glycitein and nine bound glycosides derived from them. Daidzein and genistein were the most abundant among the free glycosides. The bound glycosides are formed by three free aglycones linked with glucose, malonyl glucose, and acetyl glucose via *β*-glycosidic bond [14]. Natural soy isoflavone mainly exists in the form of non-bioactive glycosidic bonds, accounting for about 97–98%, and can be absorbed only when it is decomposed into free aglycones by *β*-glucosidase. Almost 50% of equol circulates in the free form compared to less than 20% for daidzein [15]. The deglycosylation of glycosides is an important step in isoflavone absorption and metabolism, and the metabolism of soy isoflavone is divided into 2 stages: phase I metabolism and phase II metabolism [11]. As shown in Figure 1, free aglycones can be absorbed directly by the small intestine, either actively by the intestinal epithelium or by the intestinal mucosa through passive transport, enter the lymph, and then transfer to the blood before reaching the liver for metabolism, which is phase I metabolism. The absorbed soy isoflavone in the liver binds to glucuronic acid and sulfuric acid, most of which is converted into water-soluble substances and the rest is excreted in urine in the form of gluconic anhydride or sulphate. The absorption and utilization of soy isoflavone requires a series of de-binding and binding processes, where bound glycosides can resist digestion by the stomach and small intestine to reach the colon, which undergo intestinal microbial deglycosylation [16] to become free aglycones. There are two metabolic pathways: the first is that free aglycones enter the colon and are metabolized by *β*-glucuronate transferase (UGT) and sulfur transferase (SULT) secreted by colonic bacteria before being excreted in the feces. The second pathway is that free aglycones are secreted from the liver to bile and then hydrolyzed into the intestinal tract for enterohepatic circulation, and finally excreted with urine. This type of metabolism that leaves the liver and re-enters the gut is called phase II metabolism.

By examining the daidzein metabolites found in human urine, in the intestine the biosynthesis of daidzein to produce equol occurs through a series of continuous reduction reactions, with the phase I metabolism of daidzein producing dihydrodaidzein, which is then converted to tetrahydrodaidzein, which is further metabolized into different secondary metabolites with higher biological activity [19]. The ring is then opened to form O-DMA or deketone to form equol [11]. It has been shown that equol is further metabolized in vivo to produce six metabolites including 3-OH-equol and 6-OH-equol [20], while most equol is stable in vivo. Equol is mainly produced and absorbed in the colon; the transport time of daidzein into the colon and the microbiota to convert it to equol determine the appearance of equol in plasma approximately 8 h after isoflavone ingestion [21]. According to Setchell et al. [22], plasma equol concentrations in humans following a single oral dosage of 25 mg were at their peak after 4~6 h and had an 8.8 h half-life. This was similar to other isoflavone pharmacokinetic profiles, but the plasma clearance rate of equol (CI/F = 6.85 L/h) was significantly lower than that of soy isoflavone (CI/F = 17.5 L/h). It has high bioavailability and fast absorption characteristics—a small amount of equol supplement can play a large role in the body. However, the content of equol also varies between individuals. It is currently believed that when log_10_ (equol/daidzein) > 1.75 in the human urine, it is referred to as “equol producers” and vice versa as “non-equol producers”. Studies have found that most non-equol producers convert daidzein to nonestrogenically active O-DMA [23]. Walsh et al. [24] used human Caco-2 cells to mimic intestinal cells for in vitro experiments and found that most of the equol entering the cells was converted into forms with glucoside ligands and sulphate ligands, while only the free state of equol can exert its biological activity by entering the tissue through intestinal cells. It is inferred that the main reason for the different efficiency of equol utilization in different individuals lies in the presence or absence of specific enzymes and their activity.

### 3.2. Factors Affecting the Absorption of Equol

The amount and form of soy isoflavones contained in soy and their products vary depending on how they are processed. Generally, in soy products that are processed by steaming, it is not easy to cause loss of soy isoflavones, while soaking, tofu making, and alkali treatment of soy protein can cause significant loss of soy isoflavones because soy isoflavones are water-soluble substances that are easily lost with water during processing. Soaking loss is 12%, tofu production is 44%, and soy protein separation alkali extraction is 53% [25]. Isoflavones in the form of glycosides dominate in soy and soy foods, and aglycones dominate in fermented soy foods. In order to improve the bioavailability of soy isoflavones, glycosides need to be hydrolyzed into corresponding aglycones [26]. 

According to Tamura et al. [27], the greater proportion of equol producers with lactose malabsorption is due to the fact that lactase hydrolyzes soy isoflavone glycosides, which may result in more soy flavonoids reaching the colon and being further hydrolyzed and then converted to equol. It is also possible that low lactase activity affects the composition of the diet, which in turn affects the composition of the intestinal flora. Since the bioavailability of soy isoflavones in food is influenced by many factors, dietary components can affect the intake of soy isoflavones and then affect the absorption of equol, and high carbohydrate content can significantly increase the conversion efficiency of daidzein to equol [24], hydrogen, and short-chain fatty acids (SCFAs) are the main metabolites of fermented carbohydrates in the intestinal microbiota. Decroos et al. [28] found that the production of equol is largely stimulated by hydrogen and may act as an electron donor in the bioconversion reaction from daidzein to equol. Increased equol production was also found in the presence of propionate and butyrate, suggesting that a diet rich in carbohydrates stimulates equol production. Due to chronic ingestion of significant amounts of meat, Hedlund et al. [29] discovered that Caucasian men were 4.7 times more likely to produce equol than men who did not consume meat. However, there is a significant negative correlation between the proportion of fat in the diet and urinary equol excretion, and fat intake reduced the ability of the intestinal flora to synthesize equol. Therefore, we can improve the dietary structure, increase the equol metabolic efficiency, and effectively improve the utilization rate of soy and products. Many internal factors (genetic background, gut microbiota, intestinal diseases, age, gender, etc.) and external factors (soy isoflavone source, extraction method, formula, etc.) can affect the absorption of equol [11], and the bioavailability of equol largely depends on the activity of specific gut microbiota, which play a decisive role in the metabolism of soy isoflavones [30]. It is only possible to produce equol when consuming soy foods in the presence of equol-producing bacteria. Factors that affect the composition of the gut microbiota include diet, treatment with antibiotics or other drugs, etc. [31].

## 4. Production Method of Equol

### 4.1. Chemical Synthesis of Equol

Although dietary improvement can increase the production of equol, it is only effective for equol producers and can only be supplemented externally for non-equol producers. Several studies have been developed to synthesize equol using chemical methods. The chemical synthesis method is mainly based on isoflavones and chemical reagents to prepare equol synthetically using the principles of alkylation, Friedel–Crafts reaction, hydrogenation, and Witting reaction (Table 1). Muthyal et al. [32] first proposed the use of the readily available isoflavone precursor substance daidzein as a raw material, and eventually isolated equol by chromatographic splitting using transfer hydrogenation and biomimetic synthesis techniques, obtaining a yield of 61%. However, this method requires various chemical reagents such as diacetate, ammonium formate, and acetic acid, the reduction process is difficult to control, and the catalysts used in the reaction process are expensive, resulting in a substantial increase in the cost of equol preparation. Heemstra et al. [33] obtained the central structure of equol via alkylation reaction, and then used molecular Buchwald etherification to obtain a benzodihydropyran ring; this method yields 9.8% of equol in a single batch, but is complex, and the reaction process involves expensive reagents and catalysts which are not suitable for the industrial production of equol. Gharpure et al. [34] obtained 30.75% yield from the precursor using o-quinone methyl oxide and aryl-substituted enol ethers as raw materials, but the post-treatment was tedious, with many by-products, and the separation and purification of the obtained equol was difficult. It is possible to use m-methoxyphenol and p-hydroxyphenylacetic acid as raw materials to obtain equol after a four-step reaction; although this method is simple, the total yield is not high, and it is easy to produce impurities that are not easy to separate in the process of synthesizing intermediates, and it is difficult to obtain pure substances [35]. Li et al. [36] proposed a new synthesis strategy to synthesize equol using resorcinol as a raw material, which includes a series of Wittig reactions and O-alkylation to generate isoflavone intermediates. Although this strategy improves efficiency and provides pathways for these bioactive compounds, the final product equol is a racemic compound and uses reagents that are more expensive and not suitable for industrial scale-up. In order to improve the total yield of equol, Takashima et al. [37] proposed a method for the sequential synthesis of equol from ethyl L-(-)-lactate. The key to the synthesis was the allyl substitution, and the yield of S-equol was increased to 31.6% after 11 reaction steps. Steffan et al. [38] used daidzein as a raw material to obtain equol through a four-step reaction, and the yield of equol reached 44.46%, but the initial raw material daidzein was relatively expensive, which limited its industrial application. Later, Yang et al. [39] developed an efficient iridium-catalyzed asymmetric hydrogenation reaction method, and the total yield of equol reached 48.4%.

### 4.2. Microbial Preparation of Equol

In earlier studies, it was found that equol could not be detected in urine after feeding legumes to sterile animals and newborn infants that lacked a developed microflora [41], whereas incubation of soy with human fecal flora from adults that produce equol leads to the formation of S-equol, indicating the source of equol-producing bacteria in humans and animals. Wang et al. [42] isolated the first strain of Gram-negative bacilli SNU Julong732 from human feces, which can convert dihydrodaidzein (DHD) to equol under anaerobic conditions, demonstrating that the metabolism of equol in humans is directly related to intestinal microorganisms. Whereas the degree of equol metabolism depends on various factors, dietary habits are one of the important factors leading to differences in intestinal microbiota. It is reported that only 25–30% of young people in Western countries produce equol after eating foods containing soy [43], which is significantly lower than the 50~60% equol productivity reported in Asian countries such as China, Korea, and Japan [44,45], or Western vegetarianism [46]. At the same time, the host’s intestinal microbiota, lifestyle, and genetic factors affect the production of equol; however, equol production is still determined by: (1) specific equol-producing bacteria, (2) the source of substrate derived from daidzein, and (3) an intrinsic environment capable of providing redox for the reaction to occur. Decroos et al. [28] isolated a stable mixed microbial culture (EPC4) with the ability to produce equol, namely *Enterococcus faecium* EPI1, *Lactobacillus mucosae* EPI2, *Finegodia magma* EPI3, and *Veillonella* sp. strain EP. Subsequent inoculation of EPC4 into fecal microbial cultures of non-equol producers resulted in equol production. The above results show that the ability of the strain to produce equol is naturally derived and not from an external environment. Minamida et al. [47] first isolated a strain of Gram-positive bacilli do03 from the cecal contents of rats that can convert daidzein to equol; the conversion rate of equol was increased by 4.7 and 4.5 times by adding butyric acid and arginine to GAM medium, respectively. Yu et al. [48] isolated three strains (D1, D2, and D3) which could metabolize daidzein to equol from pig feces. It was also found that adding oligofructose and inulin to the culture system was beneficial to the growth of D1, and adding acetic acid and butyric acid could increase the equol yield of D1. In addition, researchers made other attempts to increase equol production, such as adding high concentrations of nonstarch polysaccharides to the medium or increasing the content of resistant starch in the medium [49].

In addition, equol-producing bacteria have been isolated from food. For example, SNR45DH-1 and SNR48DH-1 [50] isolated from stinky tofu and *Lactococcus* strain 20–92 [51] isolated from cheese were directly used in soy germ fermentation, and produced the fermented food of soy germ lactic acid bacteria containing equol—SE5-OH [52]. It is reported that equol was found in the meat and milk of animals when fed legumes [53], especially in the organic skimmed milk produced by cows fed with red clover [54]. The presence of equol was also detected in soy beverages [55] and Pueraria [56], and engineered lactic acid bacteria strains were developed to ferment soy beverages enriched in equol [57]. With the expansion of the number and range of equol-producing bacteria, the production of equol by microbial fermentation shows a good application prospect. Although the growth rate of most equol-producing strains is very low under strict anaerobic conditions, the economical and efficient production of equol by large-scale industrial fermentation using natural bacteria will bring considerable benefits in terms of product development and production creation. Furthermore, equol is found in high levels in soy and soy products, but it can also be detected in other foods such as egg yolk, cabbage, lettuce, and other foods that also contain equol [58]. The HPLC results [59] showed that iceberg lettuce contains equol 9.56 ppm, Chinese cabbage (fermented) 17.35 ppm, Chinese cabbage (unfermented) 7.18 ppm, and kimchi 4.93 ppm.

#### 4.2.1. Independent Equol-Producing Bacteria

So far, equol-producing bacteria have been isolated from humans and animals such as mice, chickens, pigs, and cattle. Although they are from different species, the 16S rRNA genes of these bacteria are highly homologous, and most of them belong to genera of the family *Coriobacteriaceae* [60]. At present, more than fifty soy isoflavone-transformed strains have been reported at home and abroad (Table 2). They are divided into three categories according to the transformation function of the isolated strains. The first category of strains only has the hydrogenation reduction function (C-2 and C-4 positions), which can reduce daidzein and genistein to dihydrodaidzein and dihydrogenistein, respectively; the second category of strains has both hydrogenation reduction and ketone group conversion with removal of the C-4 position of the substrate, which can convert daidzein and genistein into equol and 5-OH-equol, respectively; the third category of strains has the function of ring-opening conversion, which can open the ring of substrates daidzein and genistein to O-desmethylangolensin (O-DMA) and 4-hydroxyphenyl-2-propionic acid (2-HPPA), respectively, under anaerobic conditions. Hur et al. [61] first isolated a Gram-positive anaerobic strain named HGH136 from human feces capable of converting the substrate daidzein to O-DMA via ring opening. The majority of the reported soy isoflavone transformation strains are strictly anaerobic, and only a few are facultative anaerobic bacteria such as *Enterococcus hirae* AUH-HM195 isolated from the feces of brown-eared pheasant, which can only convert the substrate daidzein into O-DMA under anaerobic conditions [62]. There is also *Proteus mirabilis* LH-52, obtained from rat gut, but its ability to synthesize equol under aerobic conditions is not stable [63]. In recent years, Heng et al. [64] isolated a facultative anaerobic Gram-positive bacilli JCM 7548 from rat feces, which is 99% similar to *Lactobacillus intestinalis*. Since *L. intestinalis* is a safe edible bacterium, it is easier to obtain and purify than many other bacteria and can be used to ferment foods containing soy.

Shigeto et al. [51] successfully isolated three strains that could directly convert daidzein to equol by using feces from 20 adult men and women as samples under anaerobic conditions with brain–heart infusion medium (BHI). These three strains were *Lactococcus* 20-92, *Eubacterium* 7-430, and *Clostridium* 20-197 after biological identification, of which *Lactococcus* 20-92 was the first reported lactic acid bacteria to directly convert daidzein to equol, but the bacteria only had the ability to transform under strict anaerobic conditions. Maruo et al. [72] isolated nine Gram-positive coccobacilli strains from human feces, classified as a new genus of the *Coriobacteriaceae* family named *Adlercreutzia equolifaciens*, seven of which could convert daidzein to equol. Yokoyama et al. [73] isolated bacteria named YY7918 from humans, which were able to convert daidzein and dihydrodaidzein to equol and identified by 16S rRNA as a new species *Eggerthella sp*. in the *Coriobacteriaceae* family. Matthies et al. [63] isolated a strain of Gram-positive anaerobic bacilli MT1B8 from mice that metabolizes not only daidzein to equol but also genistein to 5-OH-equol. Later, an equol-producing bacterial strain of Gram-positive anaerobic bacilli HE8 was isolated from human feces that converts daidzein to equol, and was named as a new species of the *Slackia* genus according to its 16S rDNA sequence and related physiological and biochemical characteristics, namely *Slackia isoflavonoconvertens* HE8 [77]. These bacteria that can independently convert soy isoflavone to equol are called independent equol-producing bacteria. As research continues, bacteria associated with equol conversion are gradually isolated and identified, and the intestinal equol-producing bacteria that could not be cultured in vitro could be identified using pyrophosphate sequencing technology. The presence of equol-producing bacteria and their direct relationship with equol metabolism have been elucidated through the study of these intestinal bacteria.

#### 4.2.2. Non-Independent Equol-Producing Bacteria

**Figure 2:**
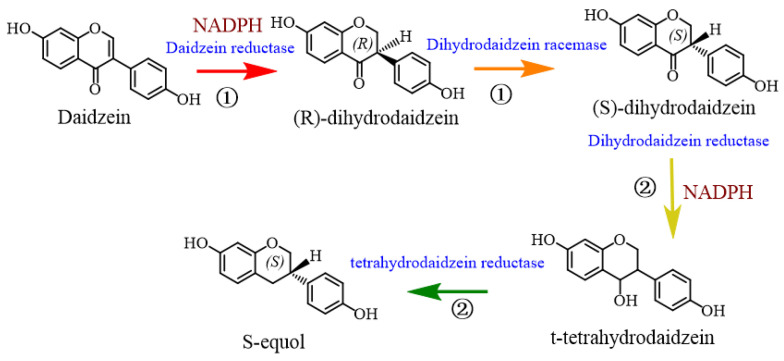
In vitro metabolic mechanism of daidzein conversion to S-equol. This schematic diagram includes four enzymes related to equol metabolism from daidzein. Among them, “①” represents bacteria involved in the first half of the reaction of equol metabolism, and “②” represents bacteria involved in the latter half of the reaction of equol metabolism.

All the strains mentioned above can directly metabolize soy isoflavone into equol. There are some other strains that only play a role in the part of the process involving the degradation of isoflavones. Some strains participate in the first half of the reaction of isoflavone metabolism to produce intermediates—dihydrodaidzein or dihydrogenistei—others can only participate in the latter half reaction of isoflavone metabolism to synthesize equol by using intermediate metabolites [8], as shown in Figure 2. Most of these bacteria are isolated from human and animal intestines, and some are pure cultures. SNU Julong732 is the first non-independent equol-producing bacterium, which can only synthesize equol by dihydrodaidzein [42]. Maruo et al. [72] also isolated two strains of Gram-positive cocci bacteria from human feces, namely FJC-A10 and FJC-A161, which can only be converted to equol by dihydrodaidzein. Hur et al. [65] screened two strains of *Escherichia coli* HGH21 and *Clostridium* HGH6 capable of metabolizing soy isoflavone from human feces, which can convert daidzin and genistin into daidzein and genistein, respectively. Among them, strain HGH6 is the first Gram-positive anaerobic bacteria with a reduction function isolated from human feces, which can further convert daidzein and genistein to dihydrodaidzein and dihydrogenistein, respectively, under anaerobic conditions. In addition, independent equol-producing bacteria were found in the feces of different animals. The strain *Pediococcus acidilactici* HXBM408 was isolated from the fresh feces of pregnant mares by Xie et al. [69], which can degrade daidzein to dihydrodaidzein. The strain *Slackia* sp. AUH-JLR41 isolated from rabbit intestine by Zhou et al. [68] was able to reduce daidzein and genistein to dihydrodaidzein and dihydrogenistein, respectively, under anaerobic conditions. Since these bacteria are only partially involved in the whole equol metabolic process, the study of their respective metabolic pathways will facilitate our better understanding of the complete mechanism of equol metabolism.

In the process of converting daidzein into equol, the researchers explore the possibility of culturing bacteria with different conversion abilities and changing the metabolic environment to achieve better results. Decroos et al. [28] identified two feces from four volunteers with the ability to degrade soy isoflavone, one with metabolic end products of dihydrodaidzein and O-DMA, and the other with dihydrodaidzein and equol. However, no single strain can transform daidzein into equol, whereas the co-culture of EPC4 produces equol. The transformation from daidzein to equol was successfully achieved by co-culturing different non-independent equol-producing bacteria. Wang et al. [91] isolated *Lactobacillus* sp. Niu-O16 from bovine rumen that converts daidzein to dihydrodaidzein and *Eggerthella* sp. Julong732 from human feces that converts dihydrodaidzein to equol, which successfully transformed into equol through inoculation on BHI broth in a mixed culture. It was also found that the combined equol production was higher than that of strain Julong732 alone. Fan et al. [92] found that the production of equol was significantly higher than that of culture alone when equol-producing anaerobic *Streptococcus faecalis* BY-1 and *Enterobacteriaceae* BY-2 were cultured at a 1:1 inoculation ratio. Jin et al. [93] converted puerarin to S-equol by co-culturing the PUE strain (which converted puerarin to daidzein) and the DZE strain (converting daidzein to equol) isolated from human feces. It can be seen that mixed cultures have more advantages than a single culture for non-independent equol-producing bacteria, and it is easier to ferment and produce equol through the synergy of different strains, thereby improving the metabolic efficiency of equol for practical application.

### 4.3. Biological Synthesis of Equol

Synthetic biology provides an efficient method for the production of equol due to substantial progress in gene sequencing and genetic engineering. At present, the main way to obtain equol is to use the selected equol-producing bacteria in vitro, using daidzein as substrate through a variety of enzymes conversion. As shown in Figure 2, four key enzymes related to equol metabolism were identified by enzyme activity analysis in vitro: daidzein reductase (DZNR), dihydrodaidzein reductase (DHDR), tetrahydrodaidzein reductase (THDR), and dihydrodaidzein racemase (DDRC) [94]. DZNR belongs to the old yellow enzyme (OYE) family and contains a 4Fe-4S iron–sulfur cluster motif (CXXCX3CX12C), two NADH/NADPH-binding motifs (GXGXXG), and an OYE-like FMN-binding domain at the N-terminal of this protein [95]. DZNR is considered to be an oxygen-sensitive enzyme with a rapid decline in activity under hyperventilation. DHDR categorized into the short-chain dehydrogenase/reductase (SDR) family, is primarily responsible for the conversion of S-dihydrodaidzein to (3S, 4R)-trans-tetrahydrodaidzein in the presence of NADPH, and is considered a rate-limiting enzyme during bioconversion of low concentrations of daidzein (less than 1 mM) [96]. THDR can only convert (3S, 4R)-trans-tetrahydrodaidzein to S-equol, which involves a free radical intermediate. THDR exhibits activity that is extremely oxygen-sensitive and is thought to be a member of the SAM (S-adenosylmethionine) enzymes [97]. Since DZNR is counterpart selective for the production of dihydrodaidzein, only S-dihydrodaidzein can be converted to S-equol, and DDRC plays an important role in the racemase reaction system for the conversion of R-dihydrodaidzein to S-dihydrodaidzein [98]. An efficient multi-enzyme cascade tool was established by introducing DZNR, DHDR, DDRC, and THDR enzymes into *E. coli* BL21 (DE3) cells, with a conversion rate of approximately 85.9% [99].

Japanese scholar Shimada et al. [100] identified the reduced coenzyme II[NADP(H)]-dependent enzymes DZNR and DHDR in the supernatant of daidzein fermentation broth from *Lactococcus* 20–92 and cloned the daidzein reductase gene *l-dznr* for the first time. The team later cloned the dihydrodaidzein reductase gene *l-dhdr* and the tetrahydrodaidzein reductase gene *l-thdr*. It was found that at least the above four key metabolic enzymes were required to convert daidzein to equol in vitro, and a mixture of the four metabolic enzymes resulted in 89.4% conversion of equol. When DDRC was missing, the conversion rate of equol was only 15.3%. DDRC with racemic enzyme activity is encoded upstream of the reductase gene in the equol cluster, which is required for efficient equol production by *Lactococcus* 20–92. Most of the biosynthetic process must be carried out under strictly anaerobic conditions and requires significant capital investment, while genetic cloning and expression of genetic mechanisms for equol production in heterologous hosts may avoid these problems [94]. Vazquez et al. [101] synthesized four key enzyme genes for equol production in *Adlercreutzia equiolifaciens* DSM19450T and cloned them into pUC57-equol vector for introduction into *E. coli*. It was proved that the recombinant *E. coli* clone could produce equol in the medium supplemented with daidzein or dihydrodaidzein. In order to overcome the problem of oxygen sensitivity of heterologous biosynthetic equol transforming bacteria, Gao et al. [102] constructed a recombinant *E. coli* with daidzein and genistein reductase genes (*dgr*) of the equol-producing bacterium *Slackia* sp. AUH-JLC159 for the first time to reduce daidzein and genistein to dihydrodaidzein and dihydrogenistein, respectively, under aerobic conditions, and the maximum transformation concentration of the two substrates was increased from 0.4 mmol/L to 1.4 mmol/L. Lee et al. [97] improved the yield of S-equol to 69.8 mg L^−1^ h^−1^ by cloning the heterologous co-expression of four enzymes converted by *Slackia isoflavoniconvertens* DSM22006 and using P212A to perform site-directed mutations of DHDR in recombinant *E. coli.* Subsequently, the team overcame the problem of low yield during fermentation using recombinant microorganisms by adding the hydrophilic polymer PVP-40K to the culture [103]. In a whole-cell biotransformation system with 5% (*w*/*v*) PVP-40k and 5% (*v*/*v*) DMSO as co-solvents, 1.22 g/L S-equol was obtained from 1.27 g/L daidzein. It is worth noting that soy isoflavone derivatives such as S-equol have an inhibitory effect on the growth and metabolism of a variety of bacteria, so when producing equol through strain metabolism, attention should be paid to the impact of equol on the metabolic bacteria. Li et al. [104] screened an S-equol resistant *E. coli* strain known as BL21 (*ydiS*) by constructing a transposon mutant library, and identified the oxidoreductase gene *ydiS* that causes resistance to S-equol for the first time, which can overcome the inhibitory effect of S-equol on bacterial growth. The above research shows that the development of synthetic biology including solvent engineering and pathway engineering provides a new idea for the large-scale production of equol. At present, the growth of the recombinant aerobic bacteria is slow, so it is necessary to further optimize the gene co-expression system and aerobic transformation process to improve the synthesis efficiency of equol.

## 5. Biological Function and Application of Equol

### 5.1. Hormone-like Effects

Equol has estrogenic activity due to its similar chemical structure to 17-*β*-estradiol, which constitutes one of the most typical biological activities of equol—estrogen-like effects. Regular intake of soy isoflavone can reduce the risk of estrogen-related diseases [12], and the incidence of breast and prostate cancer is lower in Asian countries than in Western countries due to differences in soy consumption; this may help us to understand the hypothesis that increasing the efficiency of daidzein conversion to equol after intake of soy foods by equol producer is beneficial to improving body health, namely the “equol hypothesis” [22]. Equol acts as an agonist or antagonist [105] depending on the level of endogenous estrogen, exerting a bidirectional regulatory effect with the estrogen receptor to maintain stable hormone levels in the body. The two counterparts of equol have different affinities for estrogen receptor *α* and *β* and induce endoplasmic reticulum reverse transcription activation in opposite ways, with S-equol preferentially binding to ER-*β* (Ki[ER*β*] = 16 nM; *β*/*α* = 13) and R-equol preferentially binding to ER-*α* (Ki[ER*α*] = 50 nM;; *β*/*α* = 0.29) [106], but the ability of both counterparts to bind to estrogen receptors and induce their transcriptional response is highest among isoflavone derivatives. Therefore, the differences between the effects of two nearly identical compounds on the ER subtypes are rather apparent and demonstrate how a molecule’s chiral center nicely reflects their binding affinities [107]. Further research is required to determine if these variations in ER subtype selectivity across binding and transcription experiments are caused by variations in cellular metabolism or by interactions with cellular coregulators. Equol can prevent estrogen-dependent diseases such as breast cancer, prostate cancer, osteoporosis, and so on. Mariko et al. [108] demonstrated the positive effect of S-equol on bone loss due to estrogen deficiency in ovariectomized mice, as evidenced by its ability to inhibit osteoclast formation and accelerate osteoclast apoptosis, thereby preventing osteoporosis. Huang et al. [109] reported a negative correlation between urinary equol concentrations and perimenopausal index score values in 97 perimenopausal patients, indicating that equol can significantly improve physiological and psychological symptoms. 

Equol also exhibits antiandrogenic effects by specifically binding to 5α-dihydrotestosterone (5*α*-DHT) and inhibiting its binding to the androgen receptor (AR). Epidemiological and in vitro experimental studies have shown that equol can prevent prostate cancer by reducing androgen levels and significantly reducing the proliferative ability of prostate cancer cells [110]. The anti-prostate-cancer activity of equol is associated with the activation of FOXO3a (one of the forkhead transcription factors involved in apoptosis) through protein kinase B (AKT)-specific signaling pathways, and inhibits the expression of the MDM2 complex (a negative regulator of the tumor suppressor p53) [110]. According to Stewart and Lephart [111], equol can bind to ER*β* receptors in the prostate and prevent benign prostatic hyperplasia (BPH). Meanwhile, equol stimulates estrogen-related receptor gamma (ERR-*γ*) transcription and enhances the beneficial impact of estrogen-like compounds. 

Equol can be used as a dietary supplement and can be used in place of medications to treat female estrogen insufficiency and male prostate illness. It is a successful therapy with no adverse effects [111,112]. Equol has been marketed as a nutritional supplement called “equelle”, produced by Otsuka Pharmaceutical Co., Ltd., Tokyo, Japan [113], which is widely used as an alternative to estrogen therapy for the relief of perimenopausal symptoms in menopausal women [114]. S-equol, under the trade name of AUS-131, is in the process of development against the treatment of menopausal symptoms and BPH [113].

### 5.2. Antioxidant Activity

The antioxidant activity of equol is mainly expressed in scavenging free radicals and improving antioxidant enzyme activity. As a polyphenol, the hydroxyl group in equol can be used as hydrogen donors of free radicals, which can reduce the number of free radicals and effectively inhibit low-density lipoprotein oxidation. Of all known soy isoflavone metabolites, equol is considered to be the most potent antioxidant with antioxidant activity more than 100 times higher than daidzein [115]; this may be due to the nonplanar structure of equol, which gives it greater flexibility in terms of conformation, allowing it to easily permeate the interior of the cell membrane to prevent oxidative damage to cells. The antioxidant activity of equol is mainly mediated by its interaction with ER-*β* which can induce extracellular signal-regulated protein kinases (ERK1/2) and NF-*κ*B peptides, factors that control transcription, cytokine production, and cell survival [116]. Hwang et al. [117] investigated the relationship between NO production and the antioxidant activity of equol by detecting its yield, indicating that the antioxidant activity of equol in cell culture was achieved by reducing the amount of superoxide ion (O^2−^) to increase the yield of NO. NO is known to have protective effects on arteriosclerosis and vasodilation, and its effects depend on its source and amount of production. NO produced by endogenous nitric oxide synthase (eNOS) is thought to have vasodilatory and protective effects against low-density lipoprotein into arteriosclerosis particles. In contrast, stimulation of inducible nitric oxide synthetase (iNOS) in macrophages produces high levels of NO, which has potential oxidative properties and may induce arteriosclerosis. Kang et al. [118] showed that equol was able to inhibit iNOS gene expression in macrophages by inhibiting the activation of protein kinase B (AKT) and the activity of nuclear transcription factor-*κ*B (NF-*κ*B) downstream of it. 

Equol can promote the expression of antioxidant genes in cells and enhance the activity of antioxidant enzymes, thus scavenging free radicals. Choi et al. [119] investigated the effects of equol on oxidative stress and antioxidant enzyme defense systems in the liver of mice and found that equol intake in the short term activated catalase (CAT) and superoxide dismutase (SOD) activities to resist oxidative stress. Wei et al. [120] studied the effects of equol on chicken growth and meat quality and found that equol significantly increased levels of glutathione peroxidase (GSH-px) in plasma and total superoxide dismutase (T-SOD) in pectoral muscle, which could alleviate oxidative stress and improve chicken meat quality.

### 5.3. Other Biological Functions

**Figure 3:**
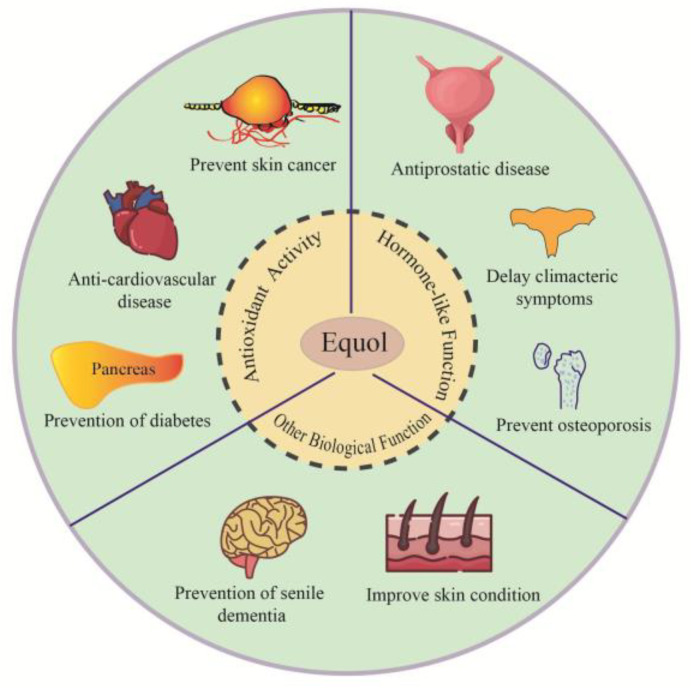
The main biological functions and applications of equol.

In addition to the above activities, the use of equol on the skin can also improve skin roughness and texture and affect skin genes including aging biomarkers proliferating cell nuclear factor, nerve growth factor, 5*α* reductase, and calcium-binding proteins S100 A8 and A9 [121]. Equol intervention studies have shown that equol reduces skin wrinkles in postmenopausal women and has anti-aging effects [122]. Moreover, equol has inhibitory effects on both matrix metalloproteinase and biomarkers of skin inflammation. These studies provide a theoretical basis for the application of equol in cosmetics and the improvement of skin health [59]. Furthermore, researchers have also found ER and AR expressions in some non-estrogen-dependent tumors, which suggest that equol can prevent non-estrogen-dependent tumors. The main biological functions of equol are shown in Figure 3.

## 6. Detection Method of Equol

With the in-depth study on the biological function of equol, the biological detection methods of equol have attracted much attention, mainly including the following common detection methods (Table 3). HPLC and gas chromatography (GC) are often used for quantitative/qualitative detection of equol, HPLC is detected by ultraviolet, fluorescent, or diode array detectors, and the purification stage uses solid phase extraction, solvent extraction, or supercritical extraction technology to carry out normal or reversed phase HPLC reactions, but the high cost of using the instrument limits its use in the laboratory [123]. Mazur et al. [124] developed a four-step procedure to extract isoflavones from food samples for GC analysis: (1) rehydration with distilled water, (2) hydrolysis with enzymes and acids, (3) purification by two ion exchange columns, and (4) derivatization. GC is usually coupled with a flame ionization detector (FID) or MS detector for detection with time-consuming hydrolysis, purification, and derivatization, but has higher specificity and sensitivity than HPLC. Liquid chromatography (LC) techniques combined with mass spectrometry (MS) is the analytical method of choice for the analysis of isoflavones in biological fluids [125]; researchers have used various internal standards as well as stable isotope labeled (SIL) analogs in combination with stable isotope dilution (SID-LC-MS) analysis [126]. At present, SID-LC-MS with selective ion monitoring is a more reliable, sensitive, and specific method for measuring equol in plasma and urine [127]. Yerramsetty et al. [128] monitored the plasma concentrations of genistein, daidzein, and their metabolite equol in rats by SID-LC-MS. Chromatographic mass spectrometry includes gas chromatography mass spectrometry (GC-MS) and liquid chromatographic mass spectrometry (LC-MS). Setchell et al. [129] used GC-MS to analyze the levels of daidzein and equol in vegetarian urine and human plasma. Elghali et al. [82] investigated the effects of Bifidobacterium breve 15700 and Bifidobacterium longum BB536 on the conversion of daidzein to equol via LC-MS. 

According to the structural characteristics of equol, it can be seen that it has an absorption peak in the ultraviolet region. However, since equol is not sensitive to ultraviolet absorption, the repeatability of the ultraviolet detection method is not high to a certain extent. Immunoassay is a highly sensitive method for equol detection, which can not only be applied to a large number of samples, but can also analyze low concentrations of soy isoflavone metabolites in complex samples [130]. Among them, fluorescent immunoassay (FIA) has advantages over radioimmunoassay (RIA) for non-radioisotope assays, such as stability, no radiation, and no waste problems, so it can be applied to routine detection work. However, enzyme immunoassay (EIA) kits currently available on the market have higher sensitivity than traditional RIA and EIA methods, and another advantage of using ELISA immunoassay techniques is the low cost of the equipment used. However, the specificity of the cross-reactivity of the antibody with equol makes it impossible to evaluate multiple isoflavone metabolites simultaneously. The ideal analytical method should have high sensitivity, low detection limits, short analysis time, high peak resolution, and good reproducibility. Single methods are often difficult to achieve these goals simultaneously, and a combination of methods is often required to achieve accurate assays.

## 7. Conclusions and Prospect

The efficient biological activity of equol has set off a research boom among scientists, it is the future research trend to explore and develop efficient and quick methods to prepare equol on a large scale and to explore more potential biological functions of equol through clinical trials. Microbial fermentation is a promising method for equol production, but problems such as slow growth rate and low equol yield need to be solved urgently. Completing the construction of engineered strains by using biosynthesis technology and optimizing the production conditions of equol is one of the effective means to improve its synthesis efficiency. In addition, equol-producing bacteria with the necessary properties can be used as probiotics in animals and human beings, and equol-related functional products can be developed to maintain health and prevent disease. At the same time, the equol-producing capacity of equol producers can be improved through diet, and the needs of non-equol producers can be met by supplementing equol or equol-producing strains. Bacterial constraints are the main barriers to industrial production and the up-scaling of equol. As a result, the development of probiotics based on safe bacteria capable of producing equol from daidzein could enable the production of equol in all individuals’ enteric environments. It is possible to use *Bifidobacterium*, *Lactococcus*, or *Lactobacillus intestinalis* with daidzein as food ingredients and supplements. Moreover, LAB strains expressing genes involved in equol production have great potential for the development of equol-enriched functional foods. Nevertheless, safety and legal issues must be considered to obtain authorization for marketing.

Although equol is effective in preventing a variety of cancers, recent studies have shown that equol can promote non-estrogen-dependent breast cancer cells, so its negative effects should not be ignored. But it is certain that equol has higher antitumor activity than its precursors, and its positive effects far outweigh the negative effects. Although equol has many nutritional benefits, uncertainty about the therapeutic mechanism of equol limits its wide application, so the effects of equol on human metabolic markers and equol production in the human body need to be clarified. With the help of metagenomics, metabolomics, and other histological analysis technologies, the metabolic pathway of equol and the molecular mechanism mediated by intestinal flora is further elucidated, so as to further study equol and promote the efficient development of equol-related industries. To fully understand the interactions within systems and how to enhance human health using a “systems biology” approach, future research must integrate data on lifestyle, genotype, gut microbiome composition and metabolome, and protein expression. 

## Figures and Tables

**Table 1:** Summary and analysis of chemical synthesis methods of equol [40].

Key Steps	Precursor	Number of Steps	Overall Yield	References
Reduction reaction	Daidzein	1	61% ^b^	[32]
Evans alkylation	Benzyl chlorides	6	9.8%	[33]
Diels–Alder reaction	o-Quinone methides and aryl-substituted enol ethers	3 ^a^	30.75% ^b^	[34]
Friedel–Crafts acylation reaction	M-methoxyphenol and p-hydroxyphenylacetic acid	4	22.3% ^b^	[35]
Wittig reaction	Resorcinol	7	31.0% ^b^	[36]
Allylic substitution	Ethyl L-(-)-lactate	11	31.6%	[37]
Reduced in a enanti-oselective manner	Daidzein	4	44.46%	[38]
Asymmetric hydrogenation	*α*-arylcinnamic acids	6	48.4%	[39]

^a^ Except the precursor prepared steps. ^b^ The product was racemic equol.

**Table 2:** Equol metabolism transformation-related strains.

Substrate	Product	Bacterial Strains	Origins	Conversion Efficiency/Time	Classifications	References
Group I						
Daidzein	DHD					
		HGH6	Human	9.3%/7 d	*Clostridium*	[65]
		Niu-O16	Bovine	100%/40 h	*Lactobacillus*	[66]
		TM-40	Human	61.1%/24 h	*Coprobacillus*	[67]
		AUH-JLR41	Rabbit	NA	*Slackia equolifaciens*	[68]
		HXBM408	Pregnant horse	NA	*Pediococcus acidilactici*	[69]
Group II						
Daidzein	Equol					
		-	Human	NA	*Bifidobacterium*	[70]
			Pure culture		*Bifidobacterium animalis*	
		EP	Human	NA	*Veillonella*	[28]
		EPI1			*Enterococcus faecium*	
		EPI3			*Finegoldia magna*	
		EPI2			*Lactobacillus mucosae*	
		AHU1763	Rat	NA	*Asaccharobacter celatus*	[47]
		zx-5, zx-7	Suzhong sows	NA	*Clostridium bifermentans*	[71]
		20-92	Human	89.4%/1 h	*Lactococcus garvieae*	[51]
		FJC-B9	Human	NA	*Adlercreutzia equolifaciens*	[72]
		YY7918	Human	100%/72 h	*Eggerthella*	[73]
		do03	Rat	17%/96 h	*Asaccharobacter celatus*	[74]
		LH-52	Rat	NA	*Proteus mirabilis*	[63]
		MT1B8	Mouse	100%/18 h	*Enterorhabdus mucosicola*	
		AUH-JLM455	Mouse	NA	*Acinetobacter* sp. (Patent)	[75]
		D1/D2	Pig	1.75%/48 h	*Eubacterium*	[48]
		JCM1123(T)	Mouse	NA	*Lactobacillus collinoides*	[76]
		HE8	Human	61.9%/14 h	*Slackia isoflavoniconvertens*	[77]
		22 strains		NA	*Bifidobacterium*	[78]
		DZE		85.6%/120 h	*Slackia equolifaciens*	[79]
		NATTS		≥90%/8 h	*Slackia*	[80]
		ATCC9338	Mouse	NA	*Lactobacillus fermentum*	[81]
		ATCC15700	Human	78.5%/96 h	*Bifidobacterium breve*	[82]
		BB536		77.2%/96 h	*Bifidobacterium longum*	
		HY-1	Human	NA	*Enterococcus faecium*	[83]
		HY-2			*Slackia isoflavoniconvert ens*	
		AUH-Julong365	Human	NA	*Eggerthella*	[84]
		SNR	Stinky tofu	12~90%/24 h	*Coriobacteriaceae*	[50]
		TM-30	Human	52%/72 h	*Coriobacteriaceae*	[85]
		C1	Chicken	NA	*Clostridium*	[86]
		CS1	Human	NA	*Pediococcus pentosaceus*	[56]
		CS2(JS1)			*Lactobacillus paracasei*	
		CS3			*Lactobacillus sakei/graminis*	
		JCM 7548	Rat	29.5%/48 h	*Lactobacillus intestinalis*	[64]
		Y11	Human	56%/120 h	*Slackia equiolifaciens*	[87]
DHD	Equol					
		SNU Julong 732	Human	>80%/96 h	*Eggerthella*	[42]
		FJC-A10/FJC-A161	Human	NA	*Adlercreutzia equolifaciens*	[72]
Group III						
Daidzein	O-DMA					
		HGH 136	Human	NA	*Clostridium*	[61]
		wK1	Human	NA	*Eubacterium ramulus*	[88]
		AUH-HM195	Brown pheasant	NA	*Enterococcus hirae*	[62]
		AUH-JLC108	Chicken	80%/24 h	*Clostridium*	[89]
		AUH-JLC140	Chicken	NA	*Clostridium*	[90]

NA: Not available. “-”: Not mentioned.

**Table 3:** Main detection methods of equol.

Detection Methods	Principle	Advantages	Disadvantages
HPLC	Separation is carried out by taking advantage of the difference in the distribution of analytes with mobile and stationary phases.	High sensitivity, high flow rate, high separation efficiency, suitable for macromolecules, thermally unstable substances	Long analysis time, low resolution, and short column lifetime
MS	Analyze by measuring the mass-to-charge ratio of the tested sample ions.	Good specificity, high sensitivity and fast analysis	High cost, the complex preparation and labor-intensive sample preparation
UV-Vis	Molecules and ions in a substance are used to absorb light in its wavelength range.	Easy operation, high accuracy, fast detection speed, low measurement cost	Poor characterization and limitations of qualitative analysis
ELISA	Assay using the color displayed after the analyte reacts with the enzyme.	Simple operation, good reproducibility and high sensitivity	Sometimes nonspecific reactions occur

## Data Availability

No new data were created or analyzed in this study. Data sharing is not applicable to this article.
